# Supplementary material for: Structural insights into the elevator-type transport mechanism of a bacterial ZIP metal transporter
Source: Nat Commun. 2023 Jan 24;14:385. doi: 10.1038/s41467-023-36048-4 (PMC9873690; doi:10.1038/s41467-023-36048-4)
Supplement: Supplementary file 1 — Supplementary Information [file 41467_2023_36048_MOESM1_ESM.pdf]

Supplementary information

Structural insights into the elevator-type transport mechanism of a bacterial ZIP metal transporter

Yao Zhang[1], Yuhan Jiang[2,*], Kaifu Gao[3,*], Dexin Sui[1], Peixuan Yu[1], Min Su[4], Guowei Wei[1,3], Jian Hu[1,2,#]

[1] Department of Biochemistry and Molecular Biology,

[2] Department of Chemistry,

[3] Department of Mathematics, Michigan State University, East Lansing, MI 48824

[4] Life Sciences Institute, University of Michigan, Ann Arbor, MI 48109

[*] These authors are equally contributive to this work.

[#] To whom correspondence should be addressed: Jian Hu, hujian1@msu.edu

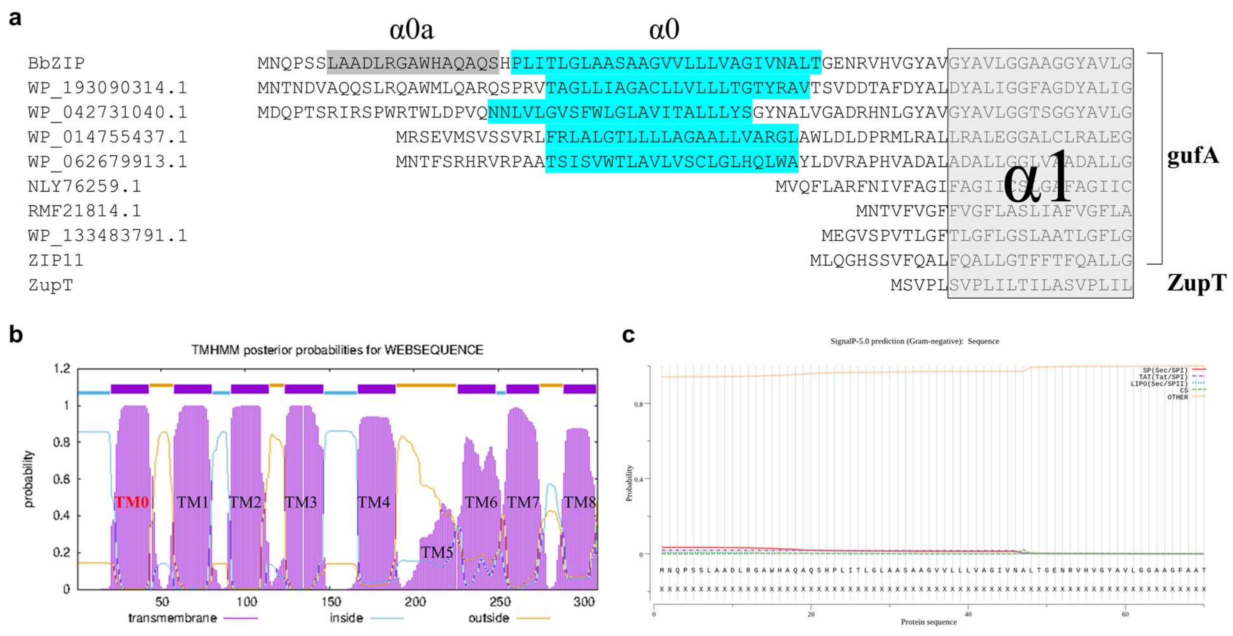

Figure S1. **Analysis of the N terminal sequence of BbZIP. a** Sequence alignment of the N terminal sequences of selected ZIPs. BbZIP, RMF21814.1 [*Cyanobacteria bacterium J083*], NLY76259.1 [*Firmicutes bacterium*], WP_133483791.1 [*Halomonas ventosae*], WP_062679913.1 [*Achromobacter denitrificans*], WP_014755437.1 [*Pseudomonas*], WP_193090314.1 [*Advenella sp. FME57*], WP_042731040.1 [*Pseudomonas*], and human ZIP11 (Q8N1S5.3) are within the gufA subfamily. ZupT from *Escherichia coli* is a representative member of the ZupT subfamily. The amphipathic helix (α0a) and α0 of BbZIP are highlighted in grey and cyan, respectively. The predicted α0 of the other ZIPs, if there is any, are also highlighted. Note that some ZIPs in the gufA subfamily do not have α0 or α0a. **b** Prediction of transmembrane helices of BbZIP by TMHMM (https://services.healthtech.dtu.dk/service.php?TMHMM-2.0). **c** Prediction of signal peptide of BbZIP by SignalP (https://services.healthtech.dtu.dk/service.php?SignalP-5.0).

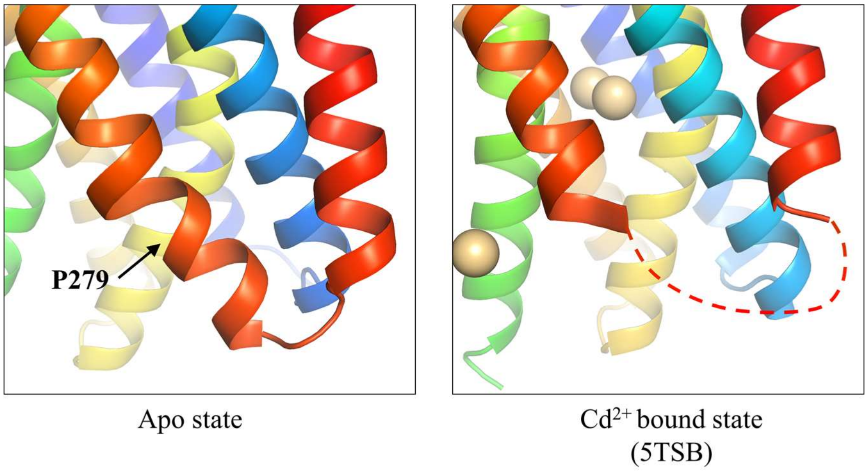

Figure S2. **Structure of the loop connecting α7 and α8.** In the apo state structure (*left*), the residues missed in the previous structure (*right*) form a broken helix and a short loop. The conserved proline residue (P279) causes a kink in α7.

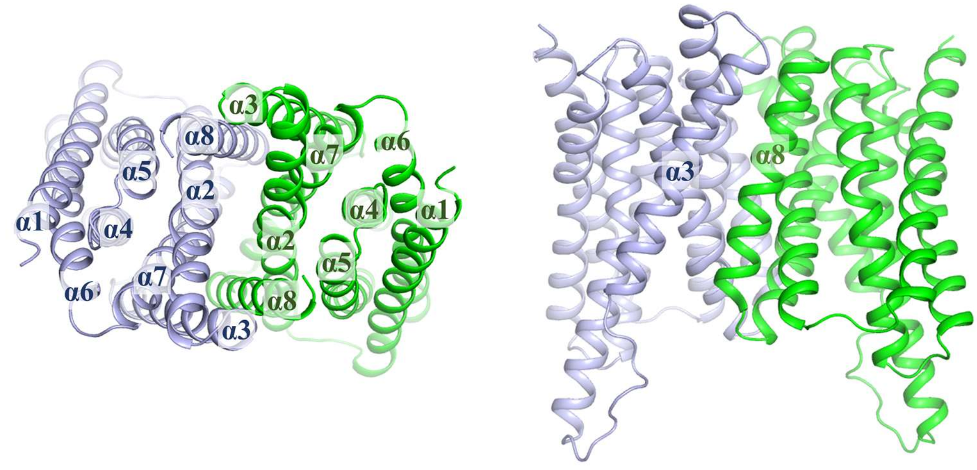

Figure S3. **BbZIP dimer predicted by AlphaFold in top view (*left*) and side view (*right*).** The structure was predicted by AlphaFold Colab.

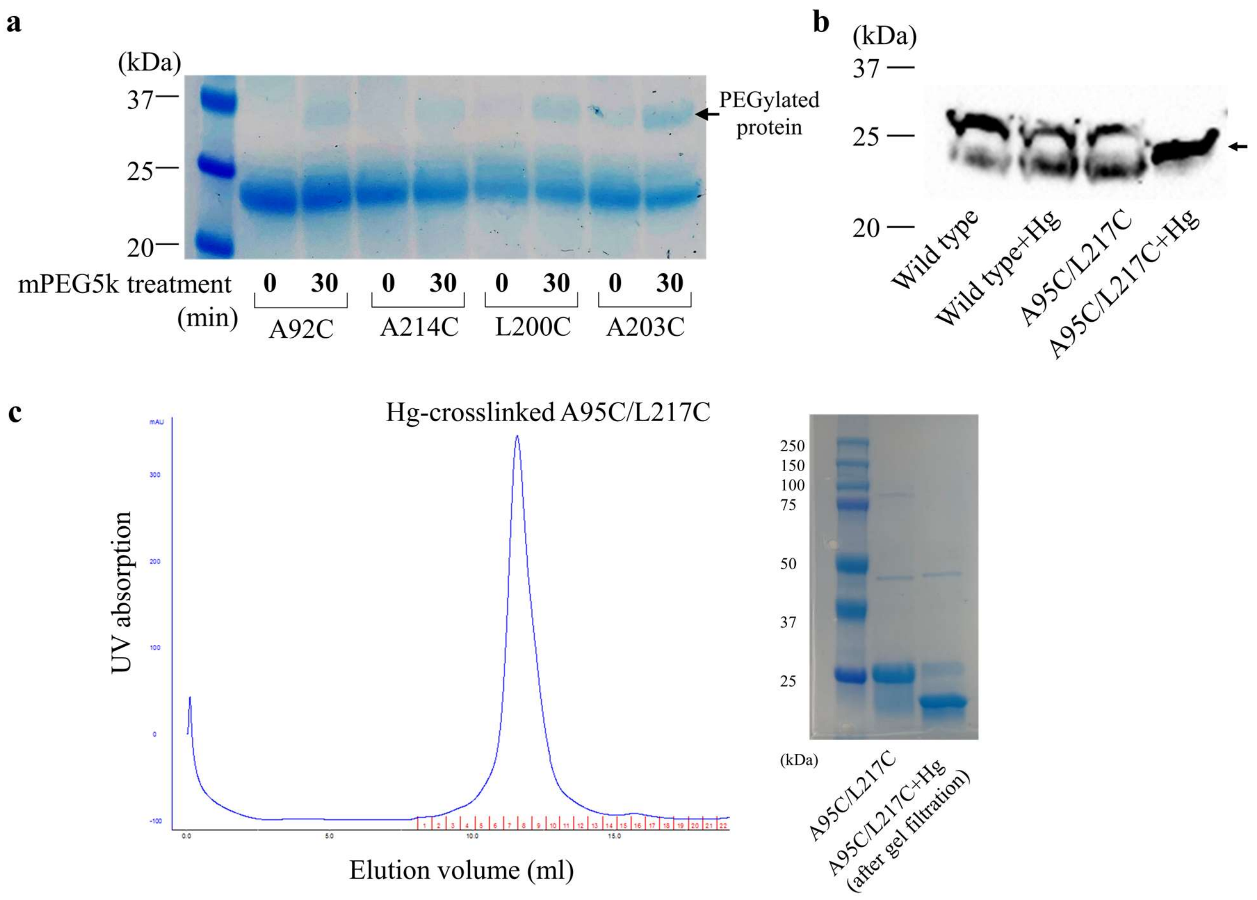

Figure S4. **Additional evidence supporting the proposed OFC model. a** Cysteine accessibility assay. Selected single cysteine variants were purified in DDM and treated with mPEG5K. EDTA was added to the sample immediately before the treatment to prevent cysteine blockage by Cd^{2+}. The reaction was terminated by 100 mM water soluble thiol reacting reagent methyl methanethiosulfonate before analysis in SDS-PAGE. The upper band indicated by the arrow is the PEGylated protein. **b** Hg-mediated chemical crosslinking of the A95C/L217C variant in the native membrane. The membrane fraction of the cells expression the variant was incubated with $HgCl_2$, terminated by NEM, and applied to Western blot by using a custom monoclonal antibody against BbZIP generated by Creative Biolabs Inc. The arrow indicates the crosslinked product. **c** Size-exclusion chromatography of the Hg-crosslinked of A95C/L217C variant. The eluted sample was applied to SDS-PAGE, and the band shift indicates that the protein is still in the crosslinked state after removal of free Hg^{2+} from the sample. Source data are provided as a Source Data file.

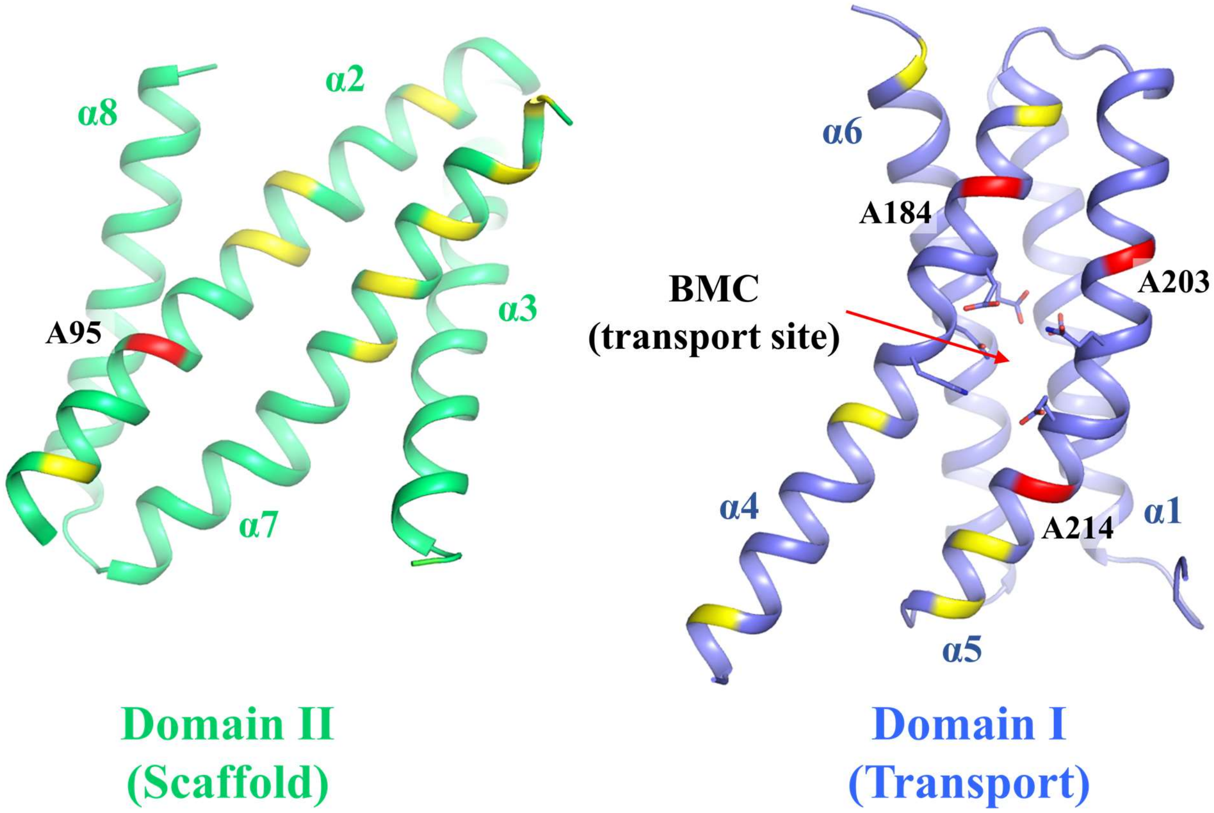

Figure S5. **Mapping of small residues at the interface between the transport domain and the scaffold domain.** The small residues (Gly, Ala, Ser) at the domain interface are colored in red (highly conserved) or yellow (less or non-conserved). The residues in the BMC (transport site) are shown in stick mode.

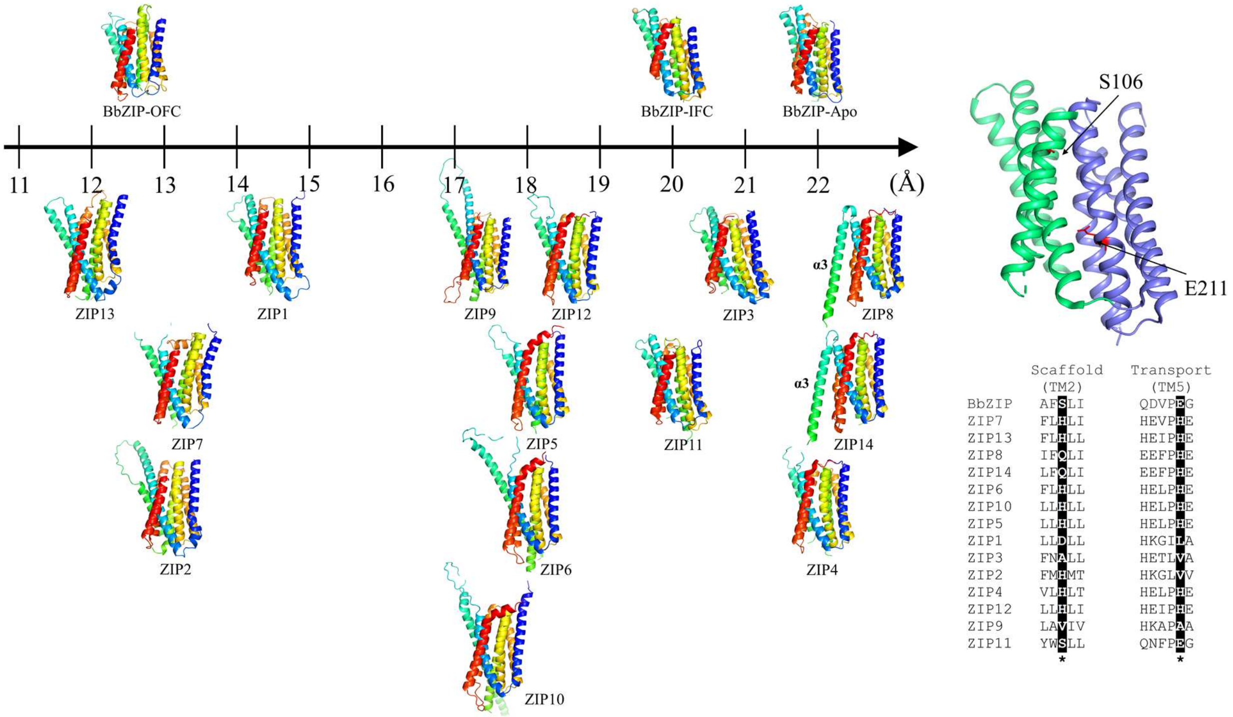

Figure S6. **Comparison of the BbZIP structures with the structures of human ZIPs predicted by AlphaFold**. All structures were retrieved from the AlphaFold Protein Structure Database (https://alphafold.ebi.ac.uk/). The scaffold domains (α2/3/7/8) of the ZIPs are structurally aligned. For clarity, the extracellular domains and the cytosolic loop between α3 and α4 are not shown. To better distinguish conformational states, the structures are plotted against the distance between a residue at the pore entrance (S106 in BbZIP) and the last residue of the metal chelating motif in α5 (E211 in BbZIP). S106 and E211 are labeled in the BbZIP structure in the apo state (right upper corner) with the scaffold domain colored in green and transport domain in blue. The residue pairs in other ZIPs for distance measurement are highlighted in the sequence alignment and indicated with asterisks. Note that α3 of ZIP8 and ZIP14 is predicted to be adjacent to α8, which is the position for α3 of the other protomer when the transporter forms a homodimer as shown in Figure 5B and Figure S3.

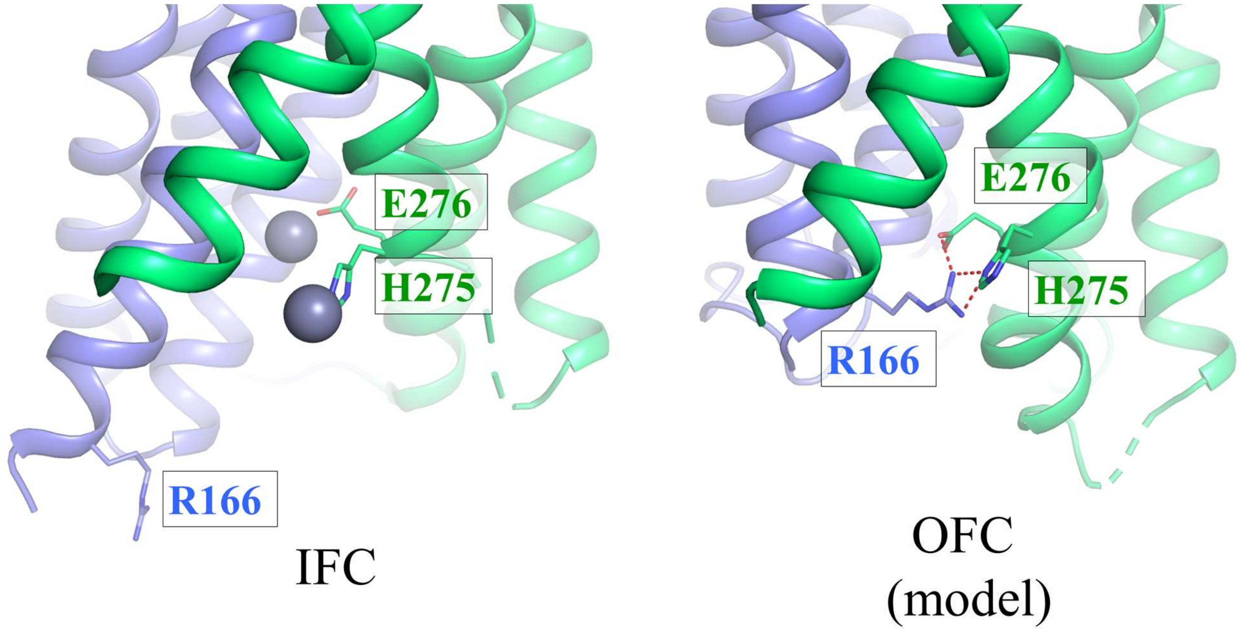

Figure S7. **A putative cytoplasmic gate in the OFC.** *Left*: IFC (PDB: 5TSA). *Right*: the OFC model. The residues involved in gate formation are labeled and shown in stick mode and. Zinc ions in the IFC are depicted as grey spheres. The hydrogen bonds are shown as red dashed lines. The transport domain and the scaffold domain are colored in blue and green, respectively.

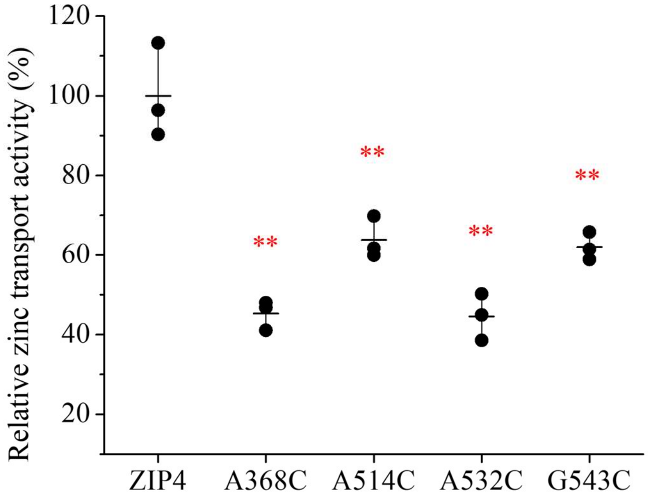

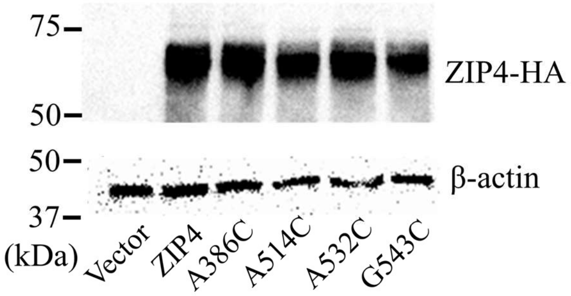

Normalized expression level

ZIP4	A368C	A514C	A532C	G543C
1.0	1.2	1.2	1.4	1.1

Figure S8. **Functional analysis of the ZIP4 variants with cysteine substitutions.** A portion of cysteine substitutions in cysteine accessibility assay and crosslinking experiments was conducted on conserved residues of BbZIP, including A95, A184, A203, and A214. The corresponding residues in human ZIP4 were replaced with cysteine and the result variants were subjected to zinc transport assay. The relative transport activity of each variant is expressed as the percentage of the activity of the wild type ZIP4. The activity has been calibrated using the expression level estimated by Western blot, which is expressed as the normalized ratio of band intensity of HA-tagged ZIP4 to β-actin. The shown data are from one representative experiment and 2-3 independent experiments with similar results were conducted for each variant. Three biological replicates were included in one experiment. The horizontal bar of the scatter dot plot represents the mean and the vertical bar indicates the standard deviation. The asterisks indicate the significant differences between the variants and the wild type ZIP4 (two-sided Student's *t*-tests: ** $P \leq 0.01$). The exact P values are 0.0016, 0.0086, 0.0019, and 0.006 for the variants of A368C, A514C, A532C, and G543C, respectively. The other residues subjected to cysteine substitution are either highly variable or have been functionally characterized in the previous reports. For instance, the functional study of H536 in human ZIP4, which is topologically equivalent to Q207 in BbZIP, has been reported in Ref 46. Source data are provided as a Source Data file.

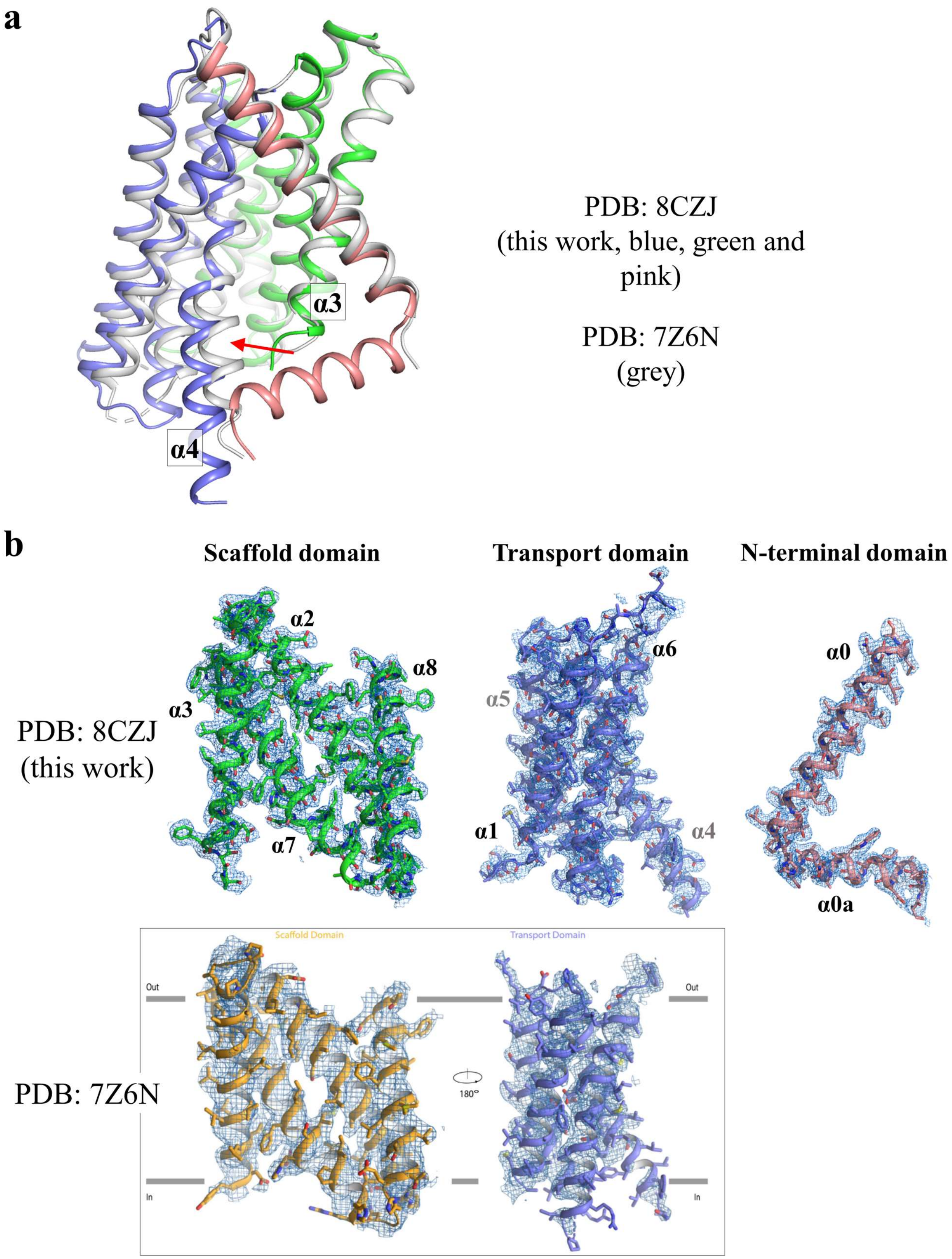
a
α3
α4
PDB: 8CZJ
(this work, blue, green and pink)
PDB: 7Z6N
(grey)
b
Scaffold domain
Transport domain
N-terminal domain
α2
α8
α3
α7
α6
α5
α1
α4
α0
α0a
PDB: 8CZJ
(this work)
Scaffold Domain
Transport Domain
Out
In
180°
PDB: 7Z6N

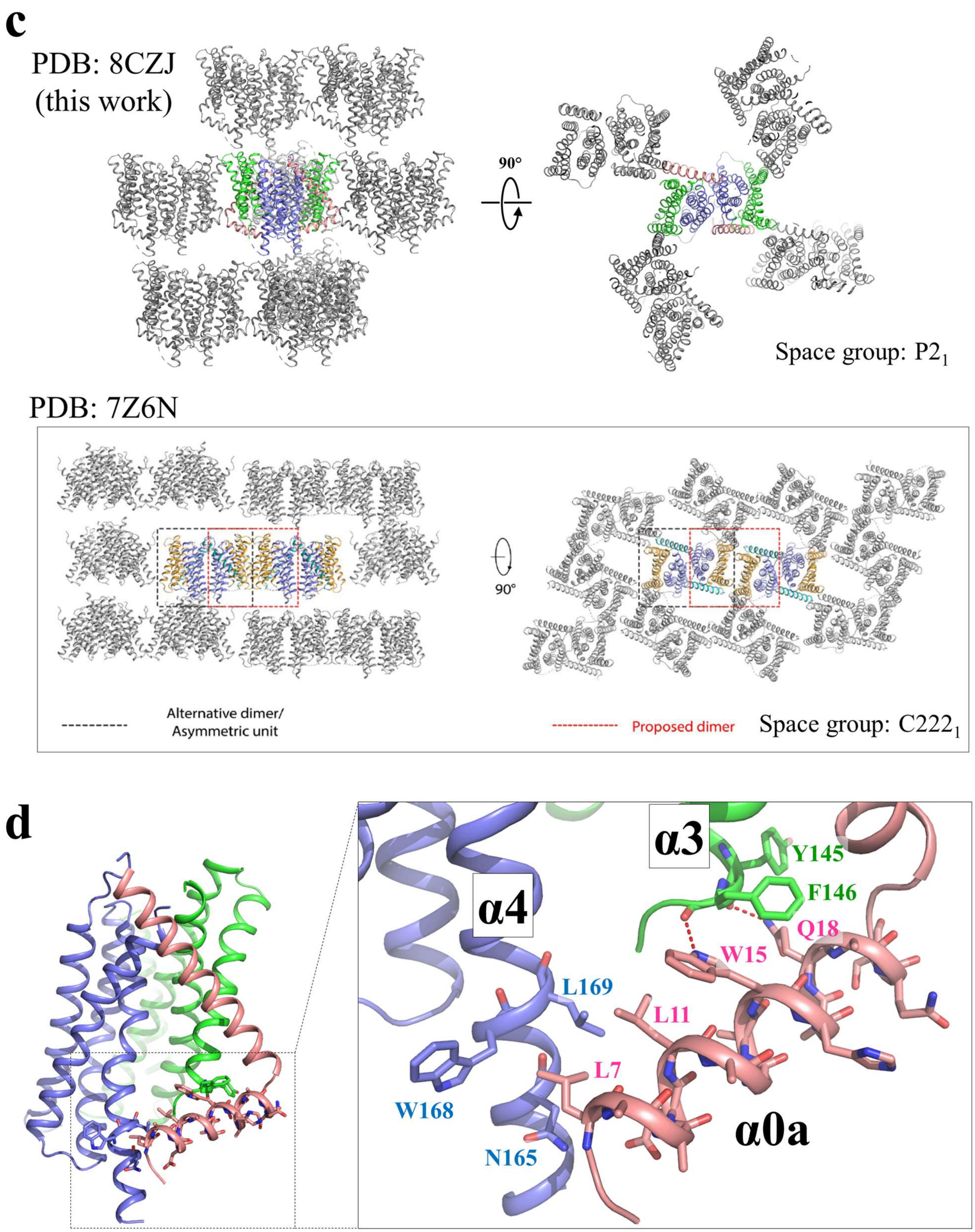

Figure S9. **Comparison of the structure solved in this work (PDB entry ID: 8CZJ) and the reported BbZIP structure in metal free state (PDB entry ID: 7Z6N). a** Structural comparison after alignment of the scaffold domains. The scaffold domain, the transport domain, and the N-

terminal domain of 8CZJ are colored in green, blue and pink, respectively. 7Z6N (chain B) is shown in grey. Note that the orientation of the transport domains relative to the scaffold domain in the two structures are different with the cytoplasmic side of α4 being the most variable region as indicated by the arrow. **b** 2FoFc electron density maps (σ=1) of 8CZJ (2.75 Å) and 7Z6N (2.6 Å). The corresponding domains are structurally aligned with labeled structural elements. The density map of the N-terminal domain of 8CZJ, including the transmembrane helix α0 and amphipathic helix α0a, is also shown. The image of 7Z6N is adapted from Fig. S1B of Ref 82. **c** Comparison of crystal packing of 8CZJ with 7Z6N. The crystallographic dimer in one asymmetry unit of 8CZJ is colored and the symmetry mates are in grey. The image of 7Z6N in frame is adapted from Fig. S4 of Ref 82. **d** Association of α0a with α3 and α4 through hydrogen bonds (red dashed lines) and hydrophobic interactions, respectively. Hypothetically, α0a may limit the elevator-like movement of the transport domain (blue) relative to the scaffold domain (green) and therefore function as a negative regulator.

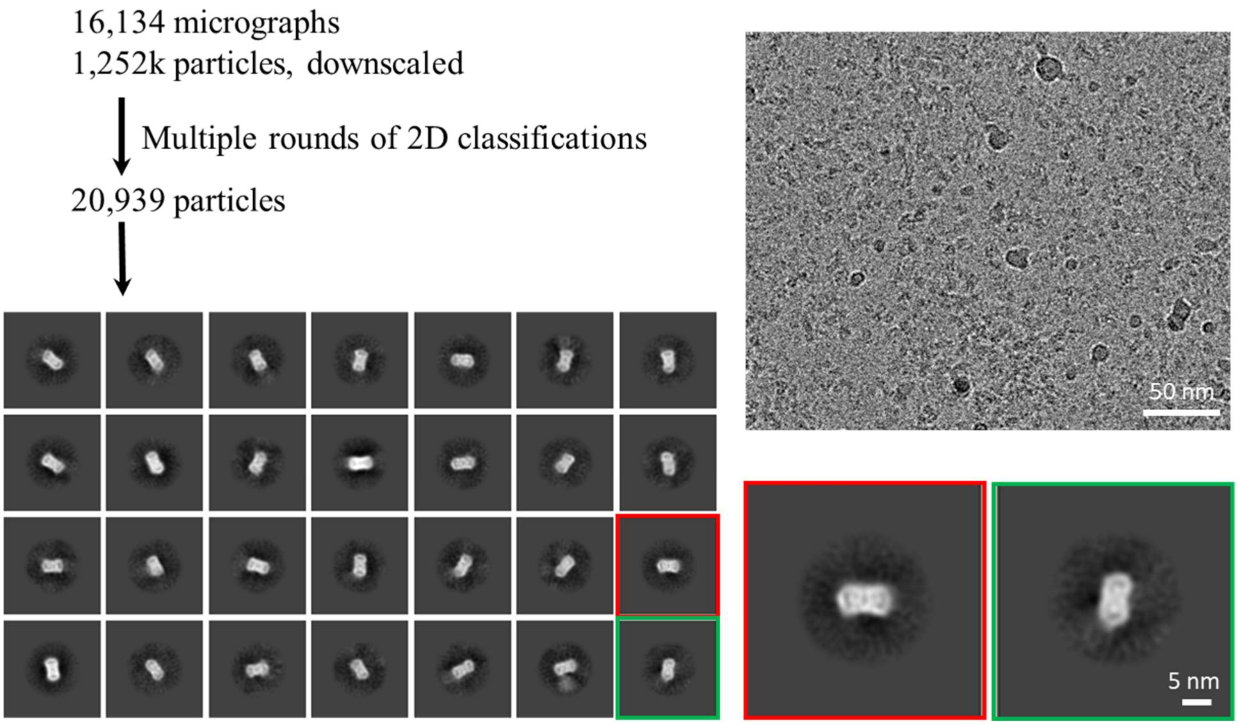

Figure S10. **The cryo-EM data processing workflow for BbZIP in Amphipol.**

a

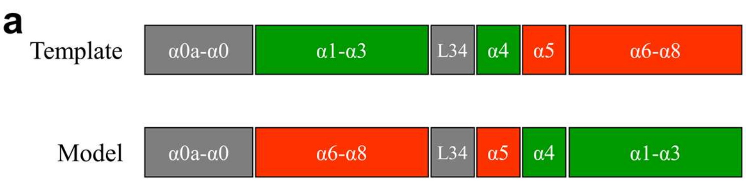

b

```
                          α0a + α0
BbZIP     MNQPSSLAADLRGAWHAQAQSHPLITLGLAASAAGVVLLLVAGIVNALTGENRV----HV
Model     MNQPSSLAADLRGAWHAQAQSHPLITLGLAASAAGVVLLLVAGIVNALTGENRVHIGRAV
          ******************************************************     *
                          α0a + α0

                 α1                                       α2
BbZIP     GYAVLGGAAGFAATALGALMALGLRAISARTQDAMLGFAAGMMLAASAFSLILPGLDAAG
Model     LVAVASGLMEPLGALVGVGISSGF----ALAYPISMGLAAGAMIFVVSHEVI-PETHR--
            ** .*     .: :*. :: *     * :    :*:*** *: . :..:* *  .
                 α6                                       α7

                       α3                        L34
BbZIP     TIVGPGPAAAAVVALGLGLGVLLMLGLDY---FTPHEHERTGHQGPEAARVNRVWLFVLT
Model     ----NG--HETTATVGLMAGFALMMFLDTALGFTPHEHERTGHQGP----LRIGLPLTSA
               *    :..::**  *. **: **    **************    :.    :. :
                       α8                        L34

          α4                                       α5
BbZIP     IILHNLPEGMAIGVSFATGD------LRIGLPLTSAIAIQDVPEGLAVALALRAVGLPIG
Model     IAIQDVPEGLAVALALRAVGLPEAARVNRVWLFVLTIILHNLPEGMAIGVSFATGD----
          * ::::***:*:.::: : .      :.    :.  :* ::::***:*:.::: : .
          α5                                       α4

                 α6                                          α7
BbZIP     RAVLVAVASGLMEPLGALVGVGISSG----FALAYPISMGLAAGAMIFVVSHEVI-PETH
Model     --VGYAVLGGAAGFAATALGALMALGLRAISARTQDAMLGFAAGMMLAASAFSLILPGLD
            *  ** .*     .: :*. :: *     * :    :*:*** *: . :..:* *  .
                 α1                                          α2

                              α8
BbZIP     R------NG--HETTATVGLMAGFALMMFLDTALG
Model     AAGTIVGPGPAAAAVVALGLGLGVLLMLGLDY---
                  *    :..::**  *. **: **
                              α3
```

Figure S11. **Sequence alignment for repeat-swap homology modeling. a** Scheme of sequence alignment of the template (BbZIP) and the model. **b** Sequence alignment of the template and the model.

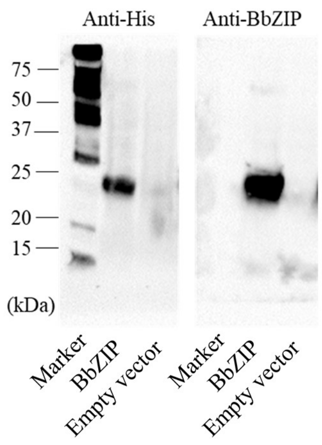

Figure S12. **Sensitivity and specificity of the custom monoclonal antibody against BbZIP.** The whole cell lysates of the cells transformed with the empty vector or the plasmid containing His_6-tagged BbZIP were applied to Western blot. The His_6-tagged BbZIP was detected with either an anti-His antibody (0.1 μg/ml) or the anti-BbZIP antibody (0.06 μg/ml). The result shows a better sensitivity and specificity of the anti-BbZIP antibody than the anti-His antibody. The shown data are from a single experiment. Source data are provided as a Source Data file.

Table S1. Crystallographic statistics

**Crystal**	Apo BbZIP
**Data collection**	
Beamline	GM/CA-CAT (23-ID-D)
Wavelength (Å)	1.0331
Space group	P 2_1
Unit cell	
a, b, c (Å)	63.0, 117.7, 64.3
α, β, γ (°)	90, 104.2, 90
[a]Resolution (Å)	37.1 - 2.75 (2.85 - 2.75)
[a]Redundancy	7.4 (2.6)
[a]Completeness (%)	94.5 (74.8)
[a]$I/\sigma I$	7.8 (0.6)
[a,b]R_{merge}	0.187 (0.968)
[a,c]R_{pim}	0.068 (0.57)
[d]$CC_{1/2}$ of the highest resolution shell	0.491
**Refinement**	
Unique reflections	22227
Number of Atoms	4289
Protein	4019
Ligands	263
H_2O	7
[e]R_{work}/R_{free}	0.233/0.261
Wilson B-factor (Å^2)	63.1
B-factors (Å^2)	
Protein	54.8
MPG	55.5
SO_4^{2-}	78.2
H_2O	52.3
R.m.s. deviations	
Bond lengths (Å)	0.010
Bond angles (°)	1.23
Ramachandran plot (%)	
Favored	97.3
Allowed	2.7
Outliers	0.0

[a]Highest resolution shell is shown in parentheses.

[b]$R_{merge} = \sum_{hkl} \sum_j |I_j(hkl) - \langle I(hkl) \rangle| / \sum_{hkl} \sum_j I_j(hkl)$, where I is the intensity of reflection.

[c]$R_{pim} = \sum_{hkl} [1/(N-1)]^{1/2} \sum_j |I_j(hkl) - \langle I(hkl) \rangle| / \sum_{hkl} \sum_j I_j(hkl)$, where N is the redundancy of the dataset.

[d]$CC_{1/2}$ is the correlation coefficient of the half datasets.

[e]$R_{work} = \sum_{hkl} | |F_{obs}| - |F_{calc}| | / \sum_{hkl} |F_{obs}|$, where F_{obs} and F_{calc} is the observed and the calculated structure factor, respectively. R_{free} is the cross-validation R factor for the test set of reflections (5% of the total) omitted in model refinement.

Table S2. Predicted contacting residues from different transmembrane helices by EVcouplings.

i	A_i	j	A_j	Probability[a]	TM	TM	# of interhelical interactions
58	Y	189	F	0.848799	1	4	3
62	G	189	F	0.998361	1	4	
66	G	185	I	0.815644	1	4	
58	Y	194	L	0.825278	1	5	14
62	G	201	T	0.988765	1	5	
65	A	205	A	0.850994	1	5	
65	A	201	T	0.801456	1	5	
69	A	205	A	0.99679	1	5	
69	A	209	V	0.96778	1	5	
72	L	209	V	0.941264	1	5	
73	G	212	G	0.77212	1	5	
74	A	216	A	0.910318	1	5	
76	M	209	V	0.951789	1	5	
76	M	213	L	0.769472	1	5	
77	A	216	A	0.822501	1	5	
78	L	216	A	0.870321	1	5	
78	L	220	R	0.784763	1	5	
59	A	244	A	0.931931	1	6	14
59	A	248	V	0.883283	1	6	
60	V	245	L	0.850106	1	6	
63	G	244	A	0.999786	1	6	
64	A	241	P	0.988794	1	6	
70	T	236	S	0.772531	1	6	
70	T	237	G	0.761363	1	6	
71	A	234	V	0.999287	1	6	
71	A	237	G	0.885252	1	6	
74	A	233	A	0.937339	1	6	
75	L	230	V	0.952012	1	6	
78	L	227	G	0.925817	1	6	
78	L	226	I	0.845856	1	6	
78	L	230	V	0.841137	1	6	
104	A	134	G	0.855523	2	3	10
108	I	133	L	0.998451	2	3	
108	I	130	A	0.778983	2	3	
111	G	129	V	0.989627	2	3	
112	L	130	A	0.998136	2	3	
112	L	129	V	0.981881	2	3	
115	A	129	V	0.94178	2	3	
115	A	128	V	0.937746	2	3	
115	A	132	G	0.891525	2	3	
116	G	127	A	0.832135	2	3	
117	T	127	A	0.906359	2	3	
95	A	218	A	0.783777	2	5	6
99	M	210	P	0.799275	2	5	
102	A	207	Q	0.930451	2	5	
102	A	200	L	0.877758	2	5	
102	A	203	A	0.82897	2	5	
106	S	207	Q	0.821258	2	5	
99	M	269	M	0.981702	2	7	28
99	M	273	V	0.932705	2	7	
100	L	273	V	0.998426	2	7	
100	L	274	S	0.983822	2	7	
100	L	270	I	0.867449	2	7	
103	S	270	I	0.996602	2	7	
103	S	269	M	0.993979	2	7	
103	S	273	V	0.965434	2	7	
103	S	266	A	0.942894	2	7	
104	A	270	I	0.987026	2	7	
107	L	262	M	0.985902	2	7	
107	L	266	A	0.968643	2	7	
108	I	266	A	0.970083	2	7	
111	G	263	G	0.999787	2	7	
111	G	259	P	0.997588	2	7	
111	G	266	A	0.989192	2	7	
114	A	259	P	0.979581	2	7	

115	A	259	P	0.988483	2	7	
85	A	284	N	0.98924	2	7	
88	Q	280	E	0.762208	2	7	
89	D	284	N	0.921114	2	7	
89	D	281	T	0.917349	2	7	
89	D	283	R	0.785205	2	7	
92	L	277	V	0.844068	2	7	
92	L	281	T	0.822336	2	7	
93	G	281	T	0.828245	2	7	
96	A	277	V	0.980601	2	7	
96	A	278	I	0.979921	2	7	
86	R	289	T	0.985952	2	8	20
89	D	290	A	0.836692	2	8	
90	A	293	G	0.999999	2	8	
90	A	289	T	0.998718	2	8	
93	G	293	G	0.939178	2	8	
93	G	290	A	0.926867	2	8	
93	G	294	L	0.90149	2	8	
94	F	300	L	0.999995	2	8	
94	F	296	A	0.954435	2	8	
94	F	297	G	0.796238	2	8	
94	F	293	G	0.771431	2	8	
96	A	294	L	0.995867	2	8	
98	M	301	M	0.931046	2	8	
100	L	294	L	0.965934	2	8	
101	A	298	F	0.981904	2	8	
101	A	301	M	0.96157	2	8	
101	A	302	M	0.812075	2	8	
102	A	301	M	0.84909	2	8	
105	F	302	M	0.971876	2	8	
106	S	301	M	0.93802	2	8	
128	V	260	I	0.940619	3	7	23
128	V	256	L	0.876523	3	7	
129	V	263	G	0.993559	3	7	
129	V	259	P	0.806656	3	7	
131	L	260	I	0.76513	3	7	
132	G	260	I	0.999996	3	7	
132	G	263	G	0.997772	3	7	
132	G	264	L	0.925151	3	7	
133	L	270	I	0.998646	3	7	
133	L	266	A	0.980342	3	7	
133	L	263	G	0.934101	3	7	
135	L	260	I	0.912802	3	7	
135	L	264	L	0.818489	3	7	
136	G	264	L	0.994045	3	7	
137	V	274	S	0.999788	3	7	
137	V	275	H	0.913608	3	7	
137	V	271	F	0.802545	3	7	
137	V	267	G	0.798816	3	7	
139	L	264	L	0.999984	3	7	
140	M	268	A	0.991156	3	7	
140	M	271	F	0.870281	3	7	
141	L	271	F	0.946312	3	7	
144	D	271	F	0.973417	3	7	
130	A	299	A	0.974493	3	8	7
134	G	299	A	0.976559	3	8	
134	G	298	F	0.907086	3	8	
137	V	295	M	0.995828	3	8	
138	L	294	L	0.912608	3	8	
138	L	298	F	0.801334	3	8	
142	G	291	T	0.958239	3	8	
167	V	222	V	0.969496	4	5	20
170	F	215	V	0.940085	4	5	
171	V	215	V	0.877697	4	5	
171	V	219	L	0.802852	4	5	
174	I	212	G	0.932936	4	5	
174	I	211	E	0.840814	4	5	
174	I	208	D	0.77545	4	5	
177	H	207	Q	0.991914	4	5	
177	H	208	D	0.750139	4	5	

178	N	208	D	0.910642	4	5	
178	N	212	G	0.888044	4	5	
181	E	208	D	0.983828	4	5	
181	E	204	I	0.90104	4	5	
181	E	211	E	0.834965	4	5	
185	I	204	I	0.980623	4	5	
185	I	201	T	0.959707	4	5	
188	S	200	L	0.996096	4	5	
188	S	197	G	0.918298	4	5	
188	S	196	I	0.910892	4	5	
189	F	197	G	0.976066	4	5	
171	V	232	V	0.990855	4	6	13
175	I	239	M	0.999986	4	6	
175	I	236	S	0.843348	4	6	
178	N	240	E	0.885684	4	6	
178	N	236	S	0.885544	4	6	
178	N	239	M	0.824308	4	6	
179	L	243	G	0.999867	4	6	
179	L	246	V	0.987151	4	6	
181	E	240	E	0.762046	4	6	
183	M	247	G	0.999611	4	6	
183	M	243	G	0.759774	4	6	
186	G	247	G	0.944232	4	6	
186	G	248	V	0.759108	4	6	
173	T	272	V	0.775614	4	7	5
183	M	261	S	0.763568	4	7	
184	A	265	A	0.783626	4	7	
186	G	265	A	0.842456	4	7	
188	S	262	M	0.759294	4	7	
208	D	240	E	0.922795	5	6	10
208	D	236	S	0.88534	5	6	
212	G	233	A	0.940804	5	6	
212	G	236	S	0.784396	5	6	
215	V	236	S	0.950061	5	6	
216	A	233	A	0.936749	5	6	
216	A	229	A	0.854505	5	6	
219	L	229	A	0.895863	5	6	
220	R	226	I	0.999554	5	6	
220	R	229	A	0.989852	5	6	
197	G	262	M	0.759117	5	7	3
208	D	269	M	0.83822	5	7	
211	E	269	M	0.924349	5	7	
274	S	294	L	0.976348	7	8	2
275	H	288	T	0.817171	7	8	

[a] Only the contacting residue pairs with probability greater than 75% are listed.

Table S3. Primers for mutagenesis in this work.

Primers	Sequences (5'-3')
**BbZIP primers**	
H55C-forward	CTG ACC GGT GAA AAT CGT GTT TGC GTT GGT TAT GCA GTT CTG GGT
H55C-reverse	ACC CAG AAC TGC ATA ACC AAC GCA AAC ACG ATT TTC ACC GGT CAG
L92C-forward	GCA CGT ACC CAG GAT GCA ATG TGC GGT TTT GCC GCA GGT ATG ATG
L92C-reverse	CAT CAT ACC TGC GGC AAA ACC GCA CAT TGC ATC CTG GGT ACG TGC
A95C-forward	CAG GAT GCA ATG CTG GGT TTT TGC GCA GGT ATG ATG CTG GCA GCC
A95C-reverse	GGC TGC CAG CAT CAT ACC TGC GCA AAA ACC CAG CAT TGC ATC CTG
A102C-forward	GCC GCA GGT ATG ATG CTG GCA TGC AGT GCA TTT AGC CTG ATT CTG
A102C-reverse	CAG AAT CAG GCT AAA TGC ACT GCA TGC CAG CAT CAT ACC TGC GGC
L138C-forward	CTG GGC CTG GGA CTG GGT GTG TGC CTG ATG CTG GGG CTG GAT TAT
L138C-reverse	ATA ATC CAG CCC CAG CAT CAG GCA CAC ACC CAG TCC CAG GCC CAG
A184C-forward	CAT AAT CTG CCG GAA GGT ATG TGC ATT GGT GTT AGC TTT GCA ACC
A184C-reverse	GGT TGC AAA GCT AAC ACC AAT GCA CAT ACC TTC CGG CAG ATT ATG
L200C-forward	GAT CTG CGT ATT GGT CTG CCG TGC ACC AGC GCC ATT GCA ATT CAG
L200C-reverse	CTG AAT TGC AAT GGC GCT GGT GCA CGG CAG ACC AAT ACG CAG ATC
A203C-forward	ATT GGT CTG CCG CTG ACC AGC TGC ATT GCA ATT CAG GAT GTT CCG
A203C-reverse	CGG AAC ATC CTG AAT TGC AAT GCA GCT GGT CAG CGG CAG ACC AAT
Q207C-forward	CTG ACC AGC GCC ATT GCA ATT TGC GAT GTT CCG GAA GGC CTG GCA
Q207C-reverse	TGC CAG GCC TTC CGG AAC ATC GCA AAT TGC AAT GGC GCT GGT CAG
A214C-forward	CAG GAT GTT CCG GAA GGC CTG TGC GTT GCC CTG GCA CTG CGT GCA
A214C-reverse	TGC ACG CAG TGC CAG GGC AAC GCA CAG GCC TTC CGG AAC ATC CTG
L217C-forward	CCG GAA GGC CTG GCA GTT GCC TGC GCA CTG CGT GCA GTG GGT CTG
L217C-reverse	CAG ACC CAC TGC ACG CAG TGC GCA GGC AAC TGC CAG GCC TTC CGG
V272C-forward	GCA GCG GGT GCA ATG ATT TTT TGC GTT AGC CAT GAA GTT ATC CCG
V272C-reverse	CGG GAT AAC TTC ATG GCT AAC GCA AAA AAT CAT TGC ACC CGC TGC
M295C- forward	ACC ACC GCA ACC GTT GGC CTG TGC GCA GGC TTT GCC CTG ATG ATG
M295C-reverse	CAT CAT CAG GGC AAA GCC TGC GCA CAG GCC AAC GGT TGC GGT GGT
**ZIP4 primers**	
A368C- forward	CTG CAG ACC TTC CTG AGC CTG TGC GTG GGT GCA CTC ACT GGG GAC
A368C- reverse	GTC CCC AGT GAG TGC ACC CAC GCA CAG GCT CAG GAA GGT CTG CAG
A368V- forward	CTG CAG ACC TTC CTG AGC CTG GTG GTG GGT GCA CTC ACT GGG GAC
A368V- reverse	GTC CCC AGT GAG TGC ACC CAC CAC CAG GCT CAG GAA GGT CTG CAG
A368F- forward	CTG CAG ACC TTC CTG AGC CTG TTT GTG GGT GCA CTC ACT GGG GAC
A368F- reverse	GTC CCC AGT GAG TGC ACC CAC AAA CAG GCT CAG GAA GGT CTG CAG
A514C- forward	CAC AAC TTC GCC GAC GGG CTG TGC GTG GGC GCC GCC TTC GCG TCC
A514C- reverse	GGA CGC GAA GGC GGC GCC CAC GCA CAG CCC GTC GGC GAA GTT GTG
A514V- forward	CAC AAC TTC GCC GAC GGG CTG GTG GTG GGC GCC GCC TTC GCG TCC
A514V- reverse	GGA CGC GAA GGC GGC GCC CAC CAC CAG CCC GTC GGC GAA GTT GTG
A514F- forward	CAC AAC TTC GCC GAC GGG CTG TTT GTG GGC GCC GCC TTC GCG TCC
A514F- reverse	GGA CGC GAA GGC GGC GCC CAC AAA CAG CCC GTC GGC GAA GTT GTG
A532C- forward	ACC GGG CTG GCC ACC TCG CTG TGC GTG TTC TGC CAC GAG TTG CCA
A532C- reverse	TGG CAA CTC GTG GCA GAA CAC GCA CAG CGA GGT GGC CAG CCC GGT
A532V- forward	ACC GGG CTG GCC ACC TCG CTG GTG GTG TTC TGC CAC GAG TTG CCA
A532V- reverse	TGG CAA CTC GTG GCA GAA CAC CAC CAG CGA GGT GGC CAG CCC GGT
A532F- forward	ACC GGG CTG GCC ACC TCG CTG TTT GTG TTC TGC CAC GAG TTG CCA
A532F- reverse	TGG CAA CTC GTG GCA GAA CAC AAA CAG CGA GGT GGC CAG CCC GGT
G543C- forward	CAC GAG TTG CCA CAC GAG CTG TGC GAC TTC GCC GCC TTG CTG CAC
G543C- reverse	GTG CAG CAA GGC GGC GAA GTC GCA CAG CTC GTG TGG CAA CTC GTG
G543V- forward	CAC GAG TTG CCA CAC GAG CTG GTG GAC TTC GCC GCC TTG CTG CAC
G543V- reverse	GTG CAG CAA GGC GGC GAA GTC CAC CAG CTC GTG TGG CAA CTC GTG
G543F- forward	CAC GAG TTG CCA CAC GAG CTG TTT GAC TTC GCC GCC TTG CTG CAC
G543F- reverse	GTG CAG CAA GGC GGC GAA GTC AAA CAG CTC GTG TGG CAA CTC GTG

Uncropped gels/Western blots

(the portions in the dashed frames are shown in Figures)

Fig. 5c

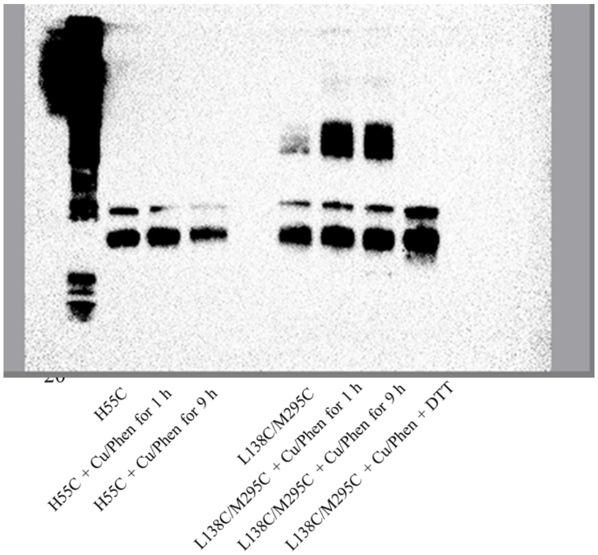

Primary: anti-Histag antibody at 1:5000 from Invitrogen, Product # 37-2900

Secondary: HRP-conjugated Horse anti-mouse IgG at 1:5000 from Cell Signaling Technology, Product # 7076S

Fig. 5d

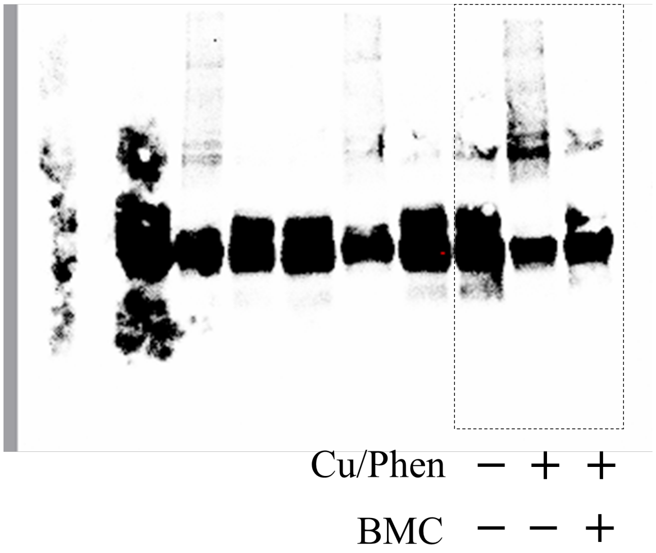

Primary: Customized anti-BbZIP monoclonal antibody at 0.06 µg/ml (generated by Creative Biolabs Inc.)

Secondary: HRP-conjugated Horse anti-mouse IgG at 1:5000 from Cell Signaling Technology, Product # 7076S

Fig. 7a

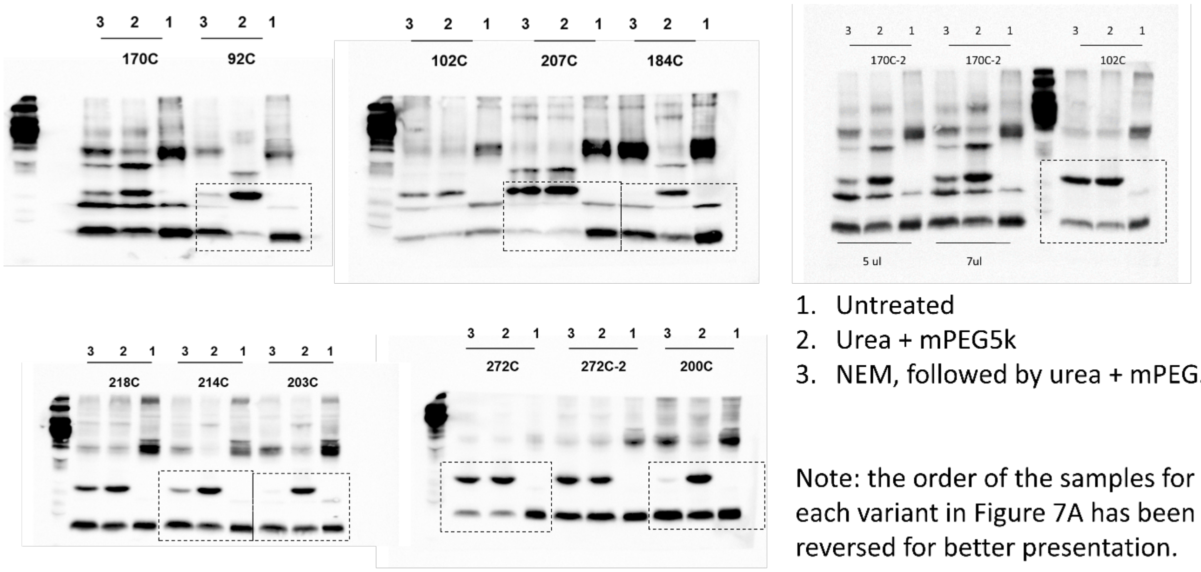

1. Untreated
2. Urea + mPEG5k
3. NEM, followed by urea + mPEG5k

Note: the order of the samples for each variant in Figure 7A has been reversed for better presentation.

Primary: anti-Histag antibody at 1:5000 from Invitrogen, Product # 37-2900

Secondary: HRP-conjugated Horse anti-mouse IgG at 1:5000 from Cell Signaling Technology, Product # 7076S

Fig. 7b

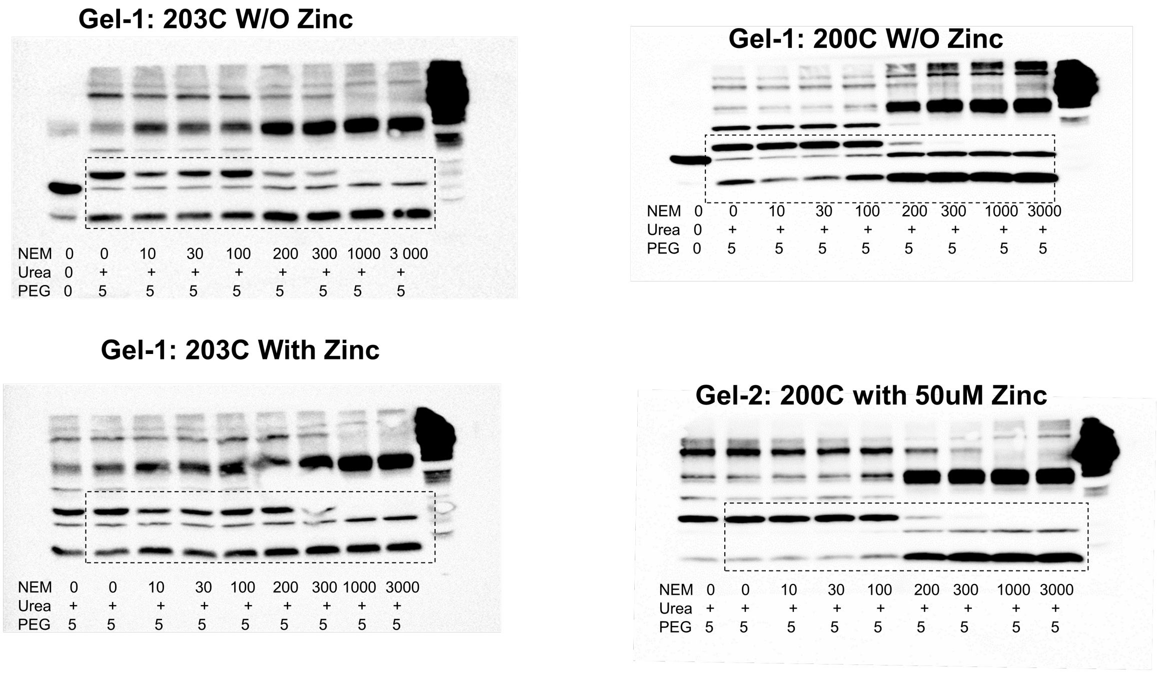

Primary: anti-Histag antibody at 1:5000 from Invitrogen, Product # 37-2900

Secondary: HRP-conjugated Horse anti-mouse IgG at 1:5000 from Cell Signaling Technology, Product # 7076S

Fig. 8

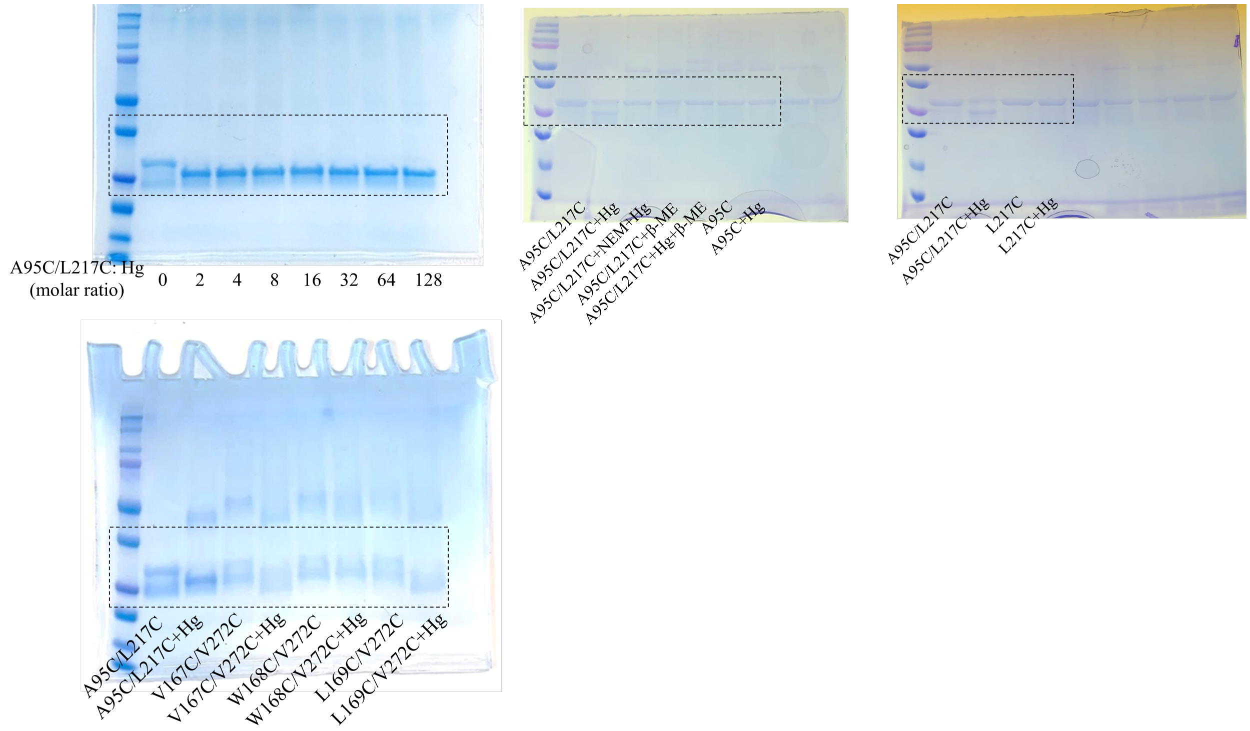
A95C/L217C: Hg
(molar ratio)
0
2
4
8
16
32
64
128
A95C/L217C
A95C/L217C+Hg
A95C/L217C+NEM+Hg
A95C/L217C+β-ME
A95C/L217C+Hg+β-ME
A95C
A95C+Hg
A95C/L217C
A95C/L217C+Hg
L217C
L217C+Hg
A95C/L217C
A95C/L217C+Hg
V167C/V272C
V167C/V272C+Hg
W168C/V272C
W168C/V272C+Hg
L169C/V272C
L169C/V272C+Hg

Fig. 9

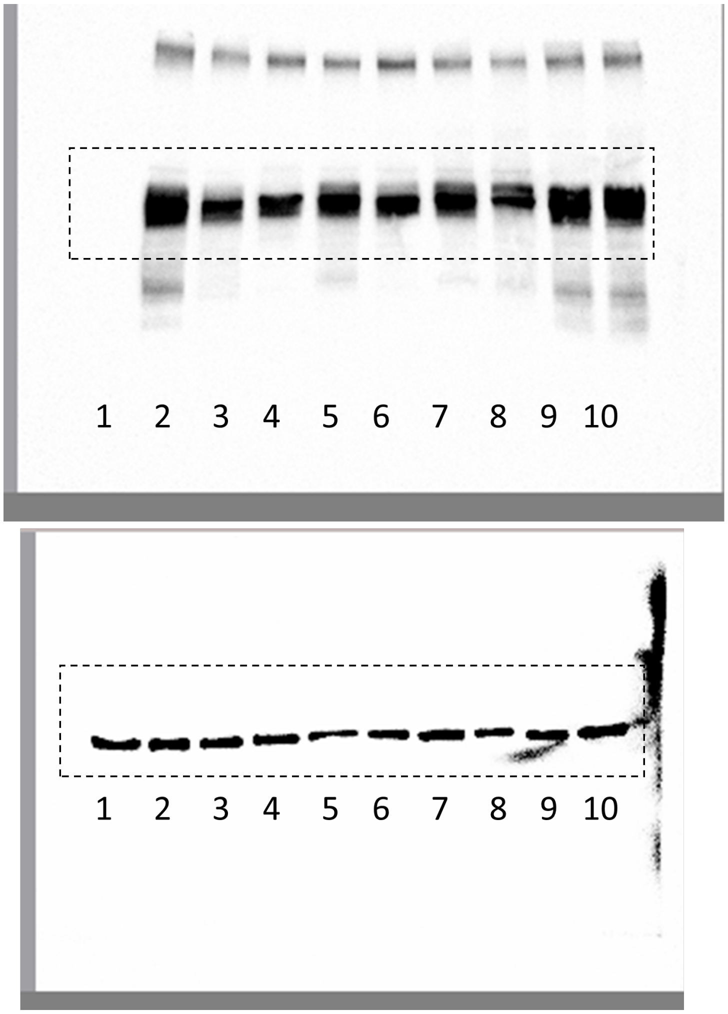

Lane1: marker and vector
Lane2: ZIP4
Lane3: 368V
Lane4: 368F
Lane5: 514V
Lane6: 514F
Lane7: 532V
Lane8: 532F
Lane9: 543V
Lane10: 543F

ZIP4-HA (upper)

Primary: anti-HA antibody at 1:5000 from Invitrogen, Product # 26183

Secondary: HRP-conjugated Horse anti-mouse IgG at 1:5000 from Cell Signaling Technology, Product # 7076S

β-actin (lower)

Primary: β-actin (13E5) Rabbit mAb at 1:5000 from Cell Signaling Technology, Product #4970S

Secondary: HRP-conjugated Goat anti-rabbit IgG at 1:5000 from Cell Signaling Technology, Product # 7074S

Supplementary Fig. 4

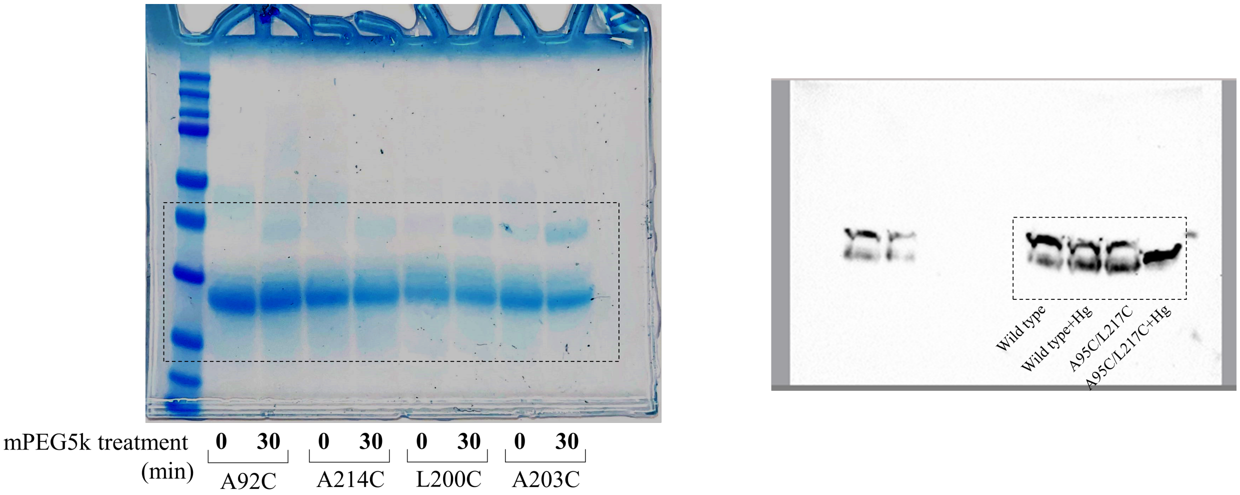

Primary: anti-Histag antibody at 1:5000 from Invitrogen, Product # 37-2900

Secondary: HRP-conjugated Horse anti-mouse IgG at 1:5000 from Cell Signaling Technology, Product # 7076S

Supplementary Fig. 8

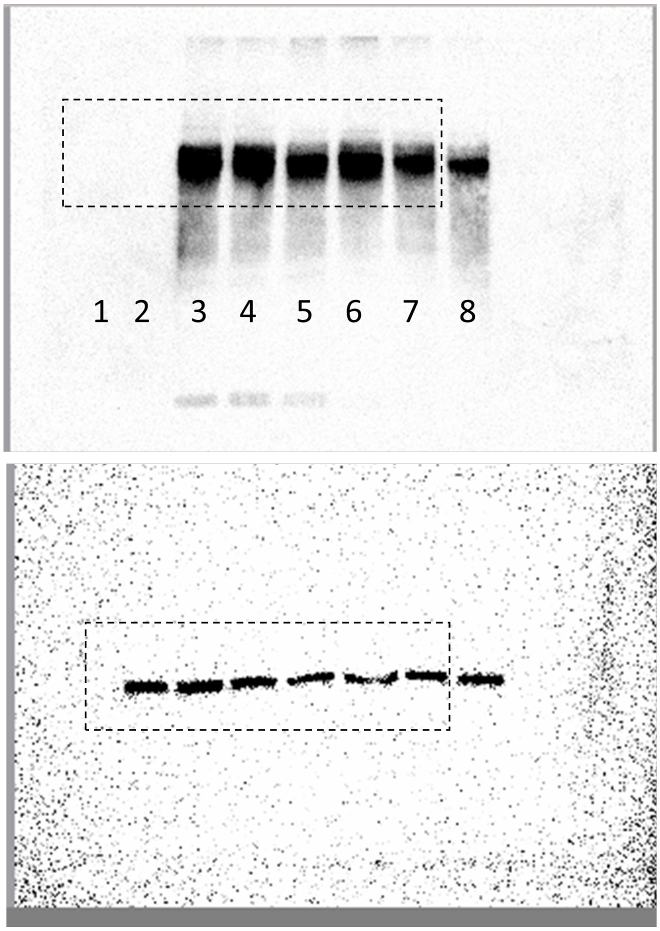

Lane1: marker
Lane2: vector
Lane3: ZIP4
Lane4: 368C
Lane5: 514C
Lane6: 532C
Lane7: 543C
Lane8: 546C

ZIP4-HA (upper)

Primary: anti-HA antibody at 1:5000 from Invitrogen, Product # 26183

Secondary: HRP-conjugated Horse anti-mouse IgG at 1:5000 from Cell Signaling Technology, Product # 7076S

β-actin (lower)

Primary: β-Actin (13E5) Rabbit mAb at 1:5000 from Cell Signaling Technology, Product #4970S

Secondary: HRP-conjugated Goat anti-rabbit IgG at 1:5000 from Cell Signaling Technology, Product # 7074S

Supplementary Fig. 12

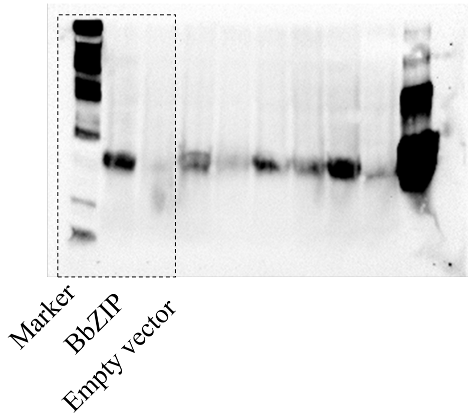

Primary: anti-Histag antibody at 0.1 µg/ml from Invitrogen, Product # 37-2900

Secondary: HRP-conjugated Horse anti-mouse IgG at 1:5000 from Cell Signaling Technology, Product # 7076S

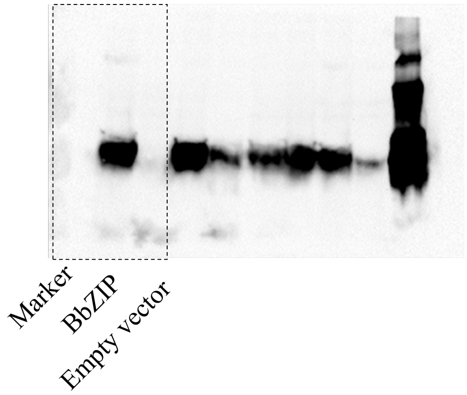

Primary: Customized anti-BbZIP monoclonal antibody at 0.06 µg/ml (generated by Creative Biolabs Inc.)

Secondary: HRP-conjugated Horse anti-mouse IgG at 1:5000 from Cell Signaling Technology, Product # 7076S
