# Supplementary material for: Structural insights into the elevator-type transport mechanism of a bacterial ZIP metal transporter
Source: Nat Commun. 2023 Jan 24;14:385. doi: 10.1038/s41467-023-36048-4 (PMC9873690; doi:10.1038/s41467-023-36048-4)
Supplement: Supplementary file 3 — Reporting Summary [file 41467_2023_36048_MOESM3_ESM.pdf]

nature portfolio

Corresponding author(s): Jian Hu

Last updated by author(s): Jan 4, 2023

Reporting Summary

Nature Portfolio wishes to improve the reproducibility of the work that we publish. This form provides structure for consistency and transparency in reporting. For further information on Nature Portfolio policies, see our Editorial Policies and the Editorial Policy Checklist.

Statistics

For all statistical analyses, confirm that the following items are present in the figure legend, table legend, main text, or Methods section.

n/a	Confirmed	
☐	☒	The exact sample size (*n*) for each experimental group/condition, given as a discrete number and unit of measurement
☐	☒	A statement on whether measurements were taken from distinct samples or whether the same sample was measured repeatedly
☐	☒	The statistical test(s) used AND whether they are one- or two-sided<br>*Only common tests should be described solely by name; describe more complex techniques in the Methods section.*
☒	☐	A description of all covariates tested
☒	☐	A description of any assumptions or corrections, such as tests of normality and adjustment for multiple comparisons
☐	☒	A full description of the statistical parameters including central tendency (e.g. means) or other basic estimates (e.g. regression coefficient) AND variation (e.g. standard deviation) or associated estimates of uncertainty (e.g. confidence intervals)
☐	☒	For null hypothesis testing, the test statistic (e.g. *F*, *t*, *r*) with confidence intervals, effect sizes, degrees of freedom and *P* value noted<br>*Give P values as exact values whenever suitable.*
☒	☐	For Bayesian analysis, information on the choice of priors and Markov chain Monte Carlo settings
☒	☐	For hierarchical and complex designs, identification of the appropriate level for tests and full reporting of outcomes
☒	☐	Estimates of effect sizes (e.g. Cohen's *d*, Pearson's *r*), indicating how they were calculated

Our web collection on statistics for biologists contains articles on many of the points above.

Software and code

Policy information about availability of computer code

Data collection	Crystallographic data were collected in synchrotron and the softwares for data collection are provided by APS.
Data analysis	HKL2000 v712, Phenix v1.10.1, COOT v0.8.2, PyMOL v1.3, cryoSPARC v3.3.2, MODELLER 9.20, AMBER v18, AlphaFold Colab

For manuscripts utilizing custom algorithms or software that are central to the research but not yet described in published literature, software must be made available to editors and reviewers. We strongly encourage code deposition in a community repository (e.g. GitHub). See the Nature Portfolio guidelines for submitting code & software for further information.

Data

Policy information about availability of data

All manuscripts must include a data availability statement. This statement should provide the following information, where applicable:

- Accession codes, unique identifiers, or web links for publicly available datasets
- A description of any restrictions on data availability
- For clinical datasets or third party data, please ensure that the statement adheres to our policy

The atomic coordinates and structure factors have been deposited in the Protein Data Bank with the access code of 8CZJ (https://www.rcsb.org/structure/unreleased/8CZJ). The structural data cited in this study are available online under PDB ID: 5TSB (https://www.rcsb.org/structure/5tsb), PDB ID: 5TSA (https://www.rcsb.org/structure/5tsa) and PDB ID: 7Z6N (https://www.rcsb.org/structure/7Z6N). All data needed to evaluate the conclusions in the paper are present in the main text and/or in the Supplementary Information. The source data underlying Figures 5c, 5d, 7a, 7b, 8a, 8b, 9c and Supplementary Figures S4a, S8, S12 are provided as a Source Data file. Additional data related to this paper may be requested from the authors.

Human research participants

Policy information about studies involving human research participants and Sex and Gender in Research.

Reporting on sex and gender	N/A
Population characteristics	N/A
Recruitment	N/A
Ethics oversight	N/A

Note that full information on the approval of the study protocol must also be provided in the manuscript.

Field-specific reporting

Please select the one below that is the best fit for your research. If you are not sure, read the appropriate sections before making your selection.

☒ Life sciences ☐ Behavioural & social sciences ☐ Ecological, evolutionary & environmental sciences

For a reference copy of the document with all sections, see nature.com/documents/nr-reporting-summary-flat.pdf

Life sciences study design

All studies must disclose on these points even when the disclosure is negative.

Sample size	In one transport assay, three biological replicates were included for each condition/variant, which follows the convention of the field, and 2-3 independent experiments were conducted for each variant/condition. For other biochemical assays, one sample was included for each condition/variant in one experiment, and 2-3 independent experiments were conducted for each condition/variant. The results of independent experiments are similar and consistent.
Data exclusions	No data were excluded unless there is strong evidence showing experiment failure, for instance, cell culture contamination.
Replication	In one transport assay, three biological replicates were included for each condition/variant, which follows the convention in the field, and 2-3 independent experiments were conducted for each variant/condition. For other biochemical assays, one sample was included for each condition/variant in one experiment, and 2-3 independent experiments were conducted for each condition/variant. The results of independent experiments are similar and consistent.
Randomization	In transport assay, the same batch of cells cultured in different subwells in the same plate behave similarly and gave consistent results. Accordingly, there is no need of randomization. For other experiments, there was no process of selecting samples from a pool of candidates, so randomization was not applicable.
Blinding	As there was no process of selecting samples from a pool of candidates, blinding cannot be applied in this work. For all the experiments presented in this study, one person is usually responsible for one type of experiment from beginning till the end. For key experiments, the results have been confirmed by a second person.

Reporting for specific materials, systems and methods

We require information from authors about some types of materials, experimental systems and methods used in many studies. Here, indicate whether each material, system or method listed is relevant to your study. If you are not sure if a list item applies to your research, read the appropriate section before selecting a response.

Materials & experimental systems

n/a	Involved in the study
	[x] Antibodies
	[x] Eukaryotic cell lines
[x]	Palaeontology and archaeology
[x]	Animals and other organisms
[x]	Clinical data
[x]	Dual use research of concern

Methods

n/a	Involved in the study
[x]	ChIP-seq
[x]	Flow cytometry
[x]	MRI-based neuroimaging

Antibodies

Antibodies used	Anti-Histag: Invitrogen, Catalog # 37-2900, Lot# WC318953<br>Anti-HA: Thermo Fisher Scientific, Cat#26183, Lot# WJ331634<br>Anti-beta actin: Cell Signaling Technology, Cat# 4970, Lot# 19<br>Custom anti-BbZIP monoclonal antibody, generated by Creative Biolabs, Inc. using purified BbZIP as antigen.<br>HRP-conjugated Horse anti-mouse IgG at 1:5000 from Cell Signaling Technology, Product # 7076S, Lot# 36<br>HRP-conjugated Goat anti-rabbit IgG at 1:5000 from Cell Signaling Technology, Product # 7074S, Lot# 30<br>Note: as this study has been conducted since 2017, the lot# of antibodies have been changed for multiple times and some records were missing. Only the Lot#s that are from the most recent experiments are reported.
Validation	Anti-Histag: Invitrogen, Catalog # 37-2900, Clone# 4A12E4. https://www.thermofisher.com/antibody/product/6x-His-Tag-Antibody-clone-4A12E4-Monoclonal/37-2900<br>Anti-HA. Thermo Fisher Scientific (Invitrogen), Cat#26183, Clone# 2-2.2.14. https://www.thermofisher.com/antibody/product/HA-Tag-Antibody-clone-2-2-2-14-Monoclonal/26183<br>Anti-beta-actin: Cell Signaling Technology, Cat# 4970, Clone# 13E5. https://www.cellsignal.com/products/primary-antibodies/b-actin-13e5-rabbit-mab/4970<br>Custom anti-BbZIP monoclonal antibody, validation data is shown in Supplementary Figure 12.

Eukaryotic cell lines

Policy information about cell lines and Sex and Gender in Research

Cell line source(s)	HEK293T from Human
Authentication	Purchased from ATCC, Catalog# CRL-3216
Mycoplasma contamination	The cell line is free of mycoplasma contamination.
Commonly misidentified lines (See ICLAC register)	HEK293T is not listed in the misidentified lines.
